# Supplementary material for: A self-complementary recombinant adeno-associated virus vector coding for an anchorless prion protein carrying the G127V mutation extends survival in a rodent prion disease model
Source: PLoS Pathog. 2026 Aug 4;22(8):e1014124. doi: 10.1371/journal.ppat.1014124 (PMC13460743; doi:10.1371/journal.ppat.1014124)

S1 Figure

A

Synthetic Bv*Prnp*^{V127}

(codon-optimized, with V127 codon shown in red and the GPI-signal sequence in green)

```
ATGGCCAACCTCAGCTACTGGCTCCTAGCTTTCTTTGTGACAACATGGACAGATGTAGGCCTGTGCAAGAAG
CGGCCAAAGCCTGGAGGCTGGAACACAGGCGGATCTAGATACCCAGGACAGGGCTCCCCTGGCGGAAACAGA
TACCCACCCCAAGGTGGTGGCACCTGGGGCCAACCCCATGGTGGTGGCTGGGGCCAACCACATGGTGGCGGT
TGGGGTCAGCCCCATGGCGGTGGCTGGGGCCAACCCCATGGTGGCGGATGGGGTCAAGGCGGCGGCACCCAC
AACCAGTGGAACAAGCCTTCCAAGCCAAAGACAAACATAAAGCATGTGGCAGGGGCTGCAGCAGCTGGGGCT
GTTGTTGGGGGCCTTGGAGTGTACATGCTGGGATCAGCCATGAGCAGGCCCATGATCCACTTTGGAAATGAC
TGGGAAGATAGATACTACAGAGAAAACATGAATAGATACCCTAACCAGGTGTACTACAGGCCAGTGGACCAG
TACAACAACCAGAACAACTTTGTCCATGACTGTGTGAACATCACAATCAAGCAGCACACAGTGACCACAACC
ACAAAAGGAGAGAACTTCACTGAGACTGATGTCAAGATGATGGAAAGAGTGGTTGAGCAGATGTGTGTCACC
CAGTACCAGAAAGAGTCCCAGGCCTACTATGAGGGCAGATCCAGCAGAGCTGTGCTGCTGTTCAGCAGCCCT
CCTGTGATCCTGCTGATCTCCTTCCTGATCTTTCTGATTGTGGGCTGA
```

B

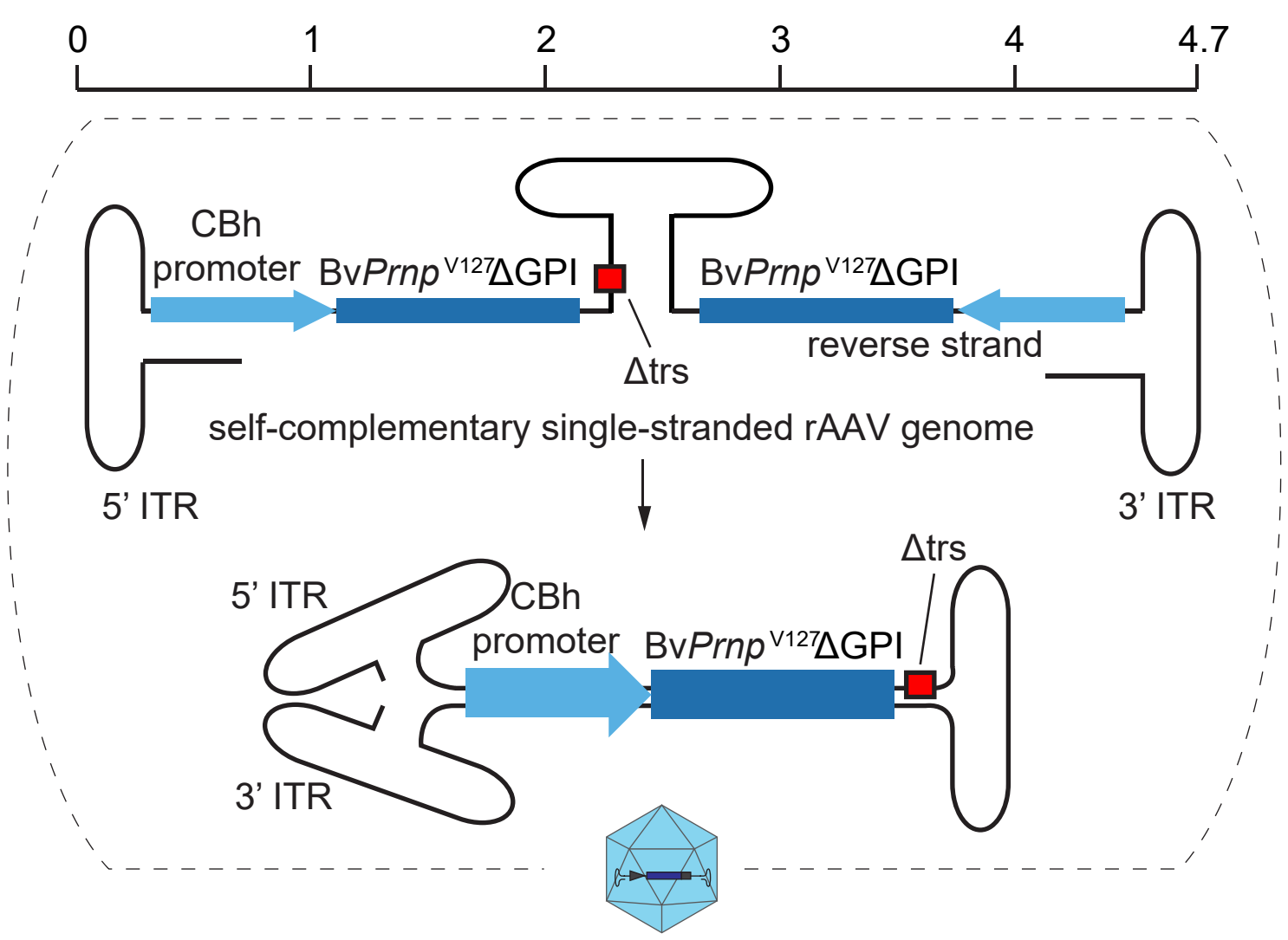

C

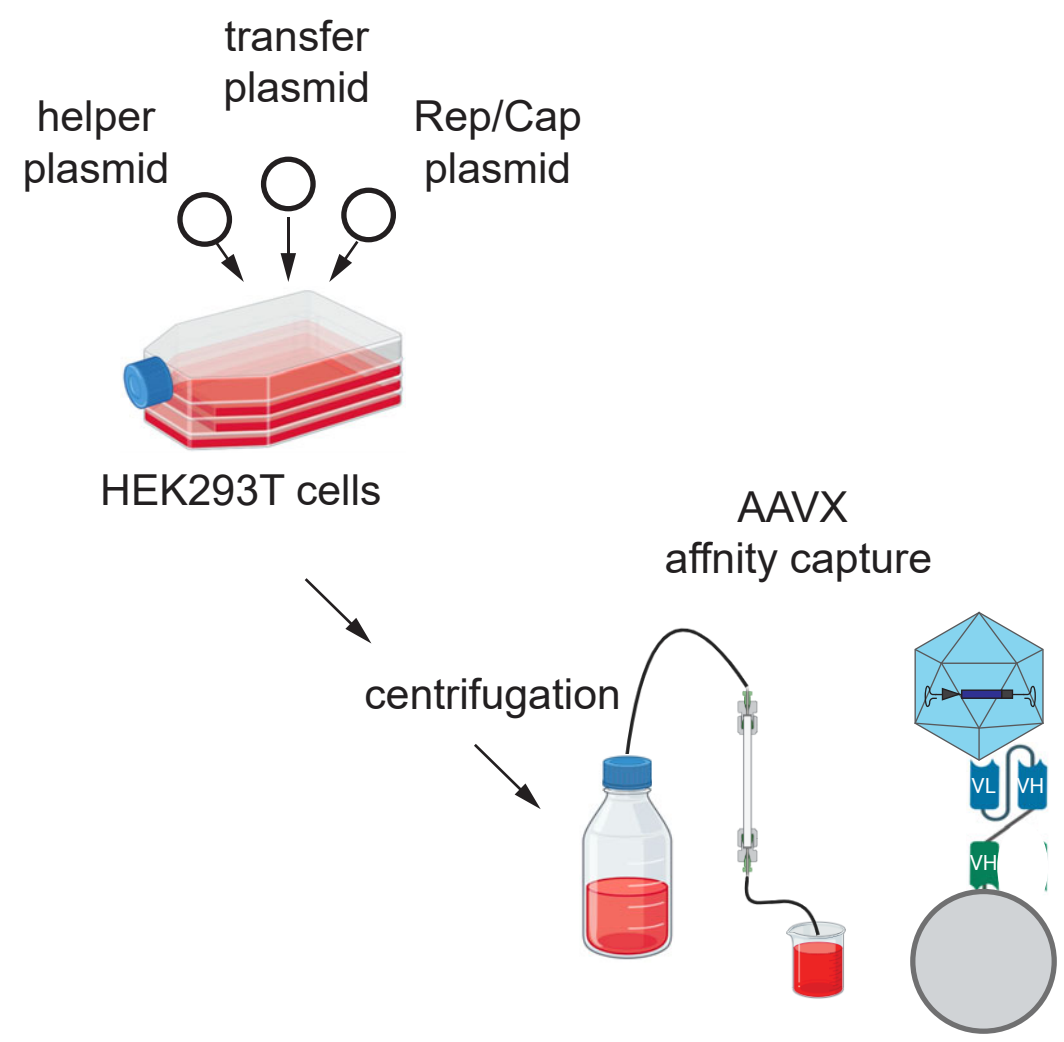

D

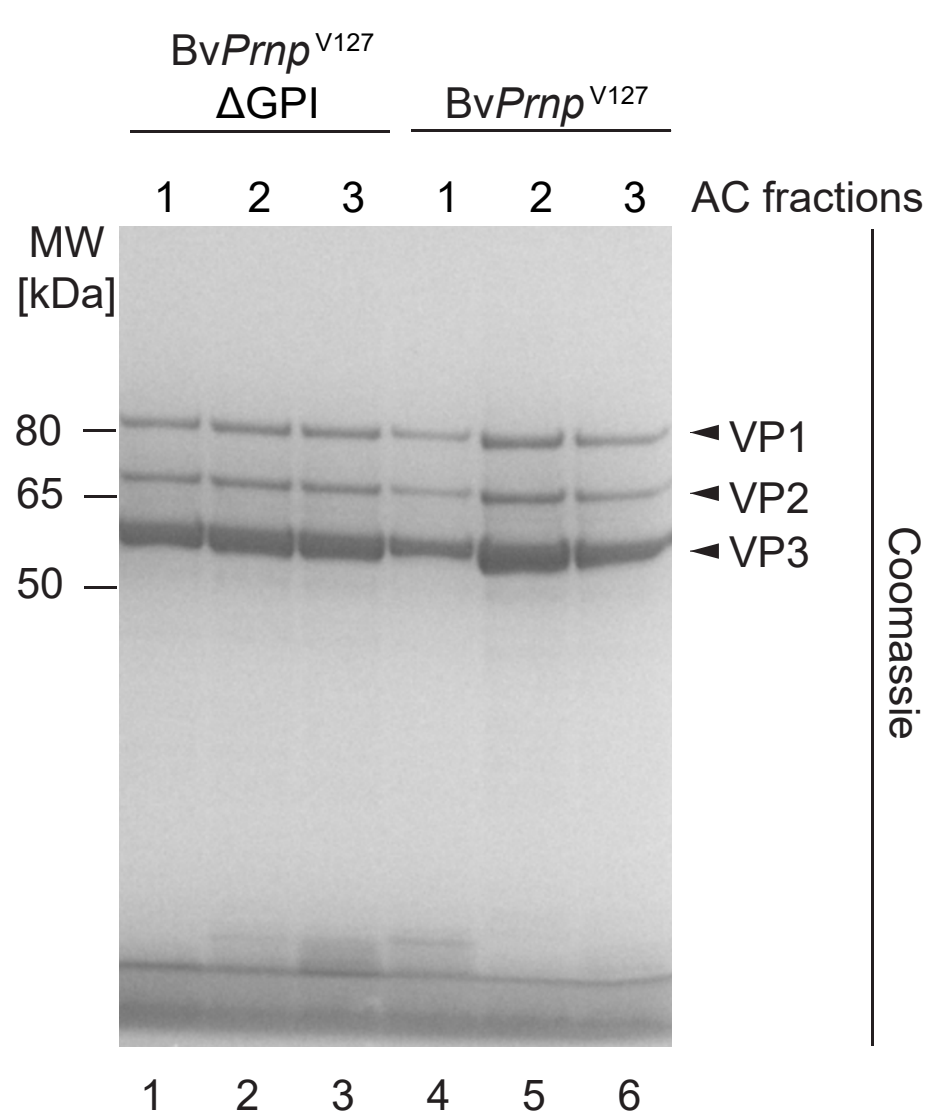

E

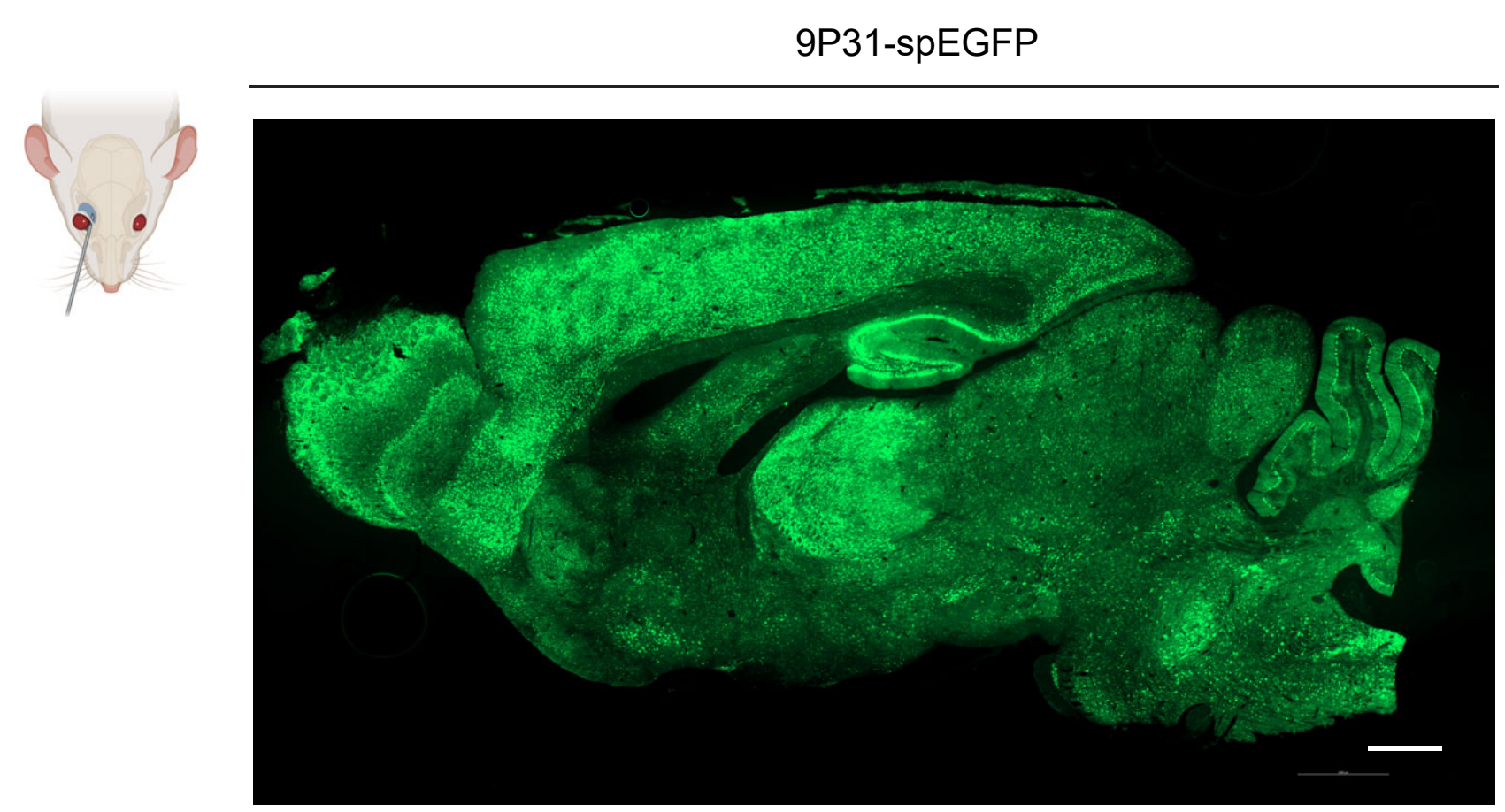

Supplement: Supplementary file 1 [file ppat.1014124.s001.pdf]
