# Supplementary material for: A self-complementary recombinant adeno-associated virus vector coding for an anchorless prion protein carrying the G127V mutation extends survival in a rodent prion disease model
Source: PLoS Pathog. 2026 Aug 4;22(8):e1014124. doi: 10.1371/journal.ppat.1014124 (PMC13460743; doi:10.1371/journal.ppat.1014124)

S2 Figure

Gfap (H&E counterstain)

Iba1 stains (H&E counterstain)

A

inoculum	preparation	capsid	payload	DPI
RML	none	none	none	152

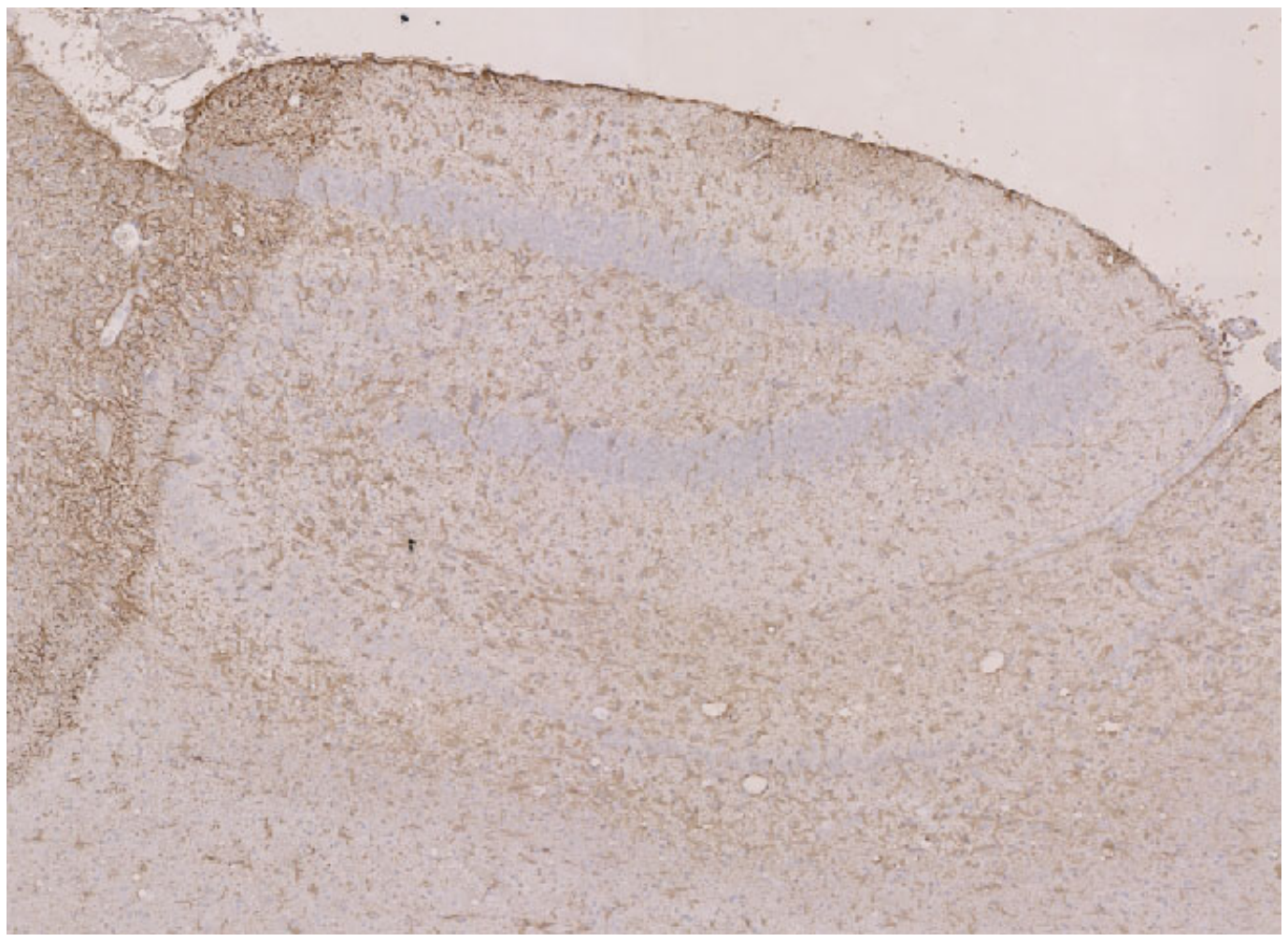

B

inoculum	preparation	capsid	payload	DPI
RML	none	none	none	152

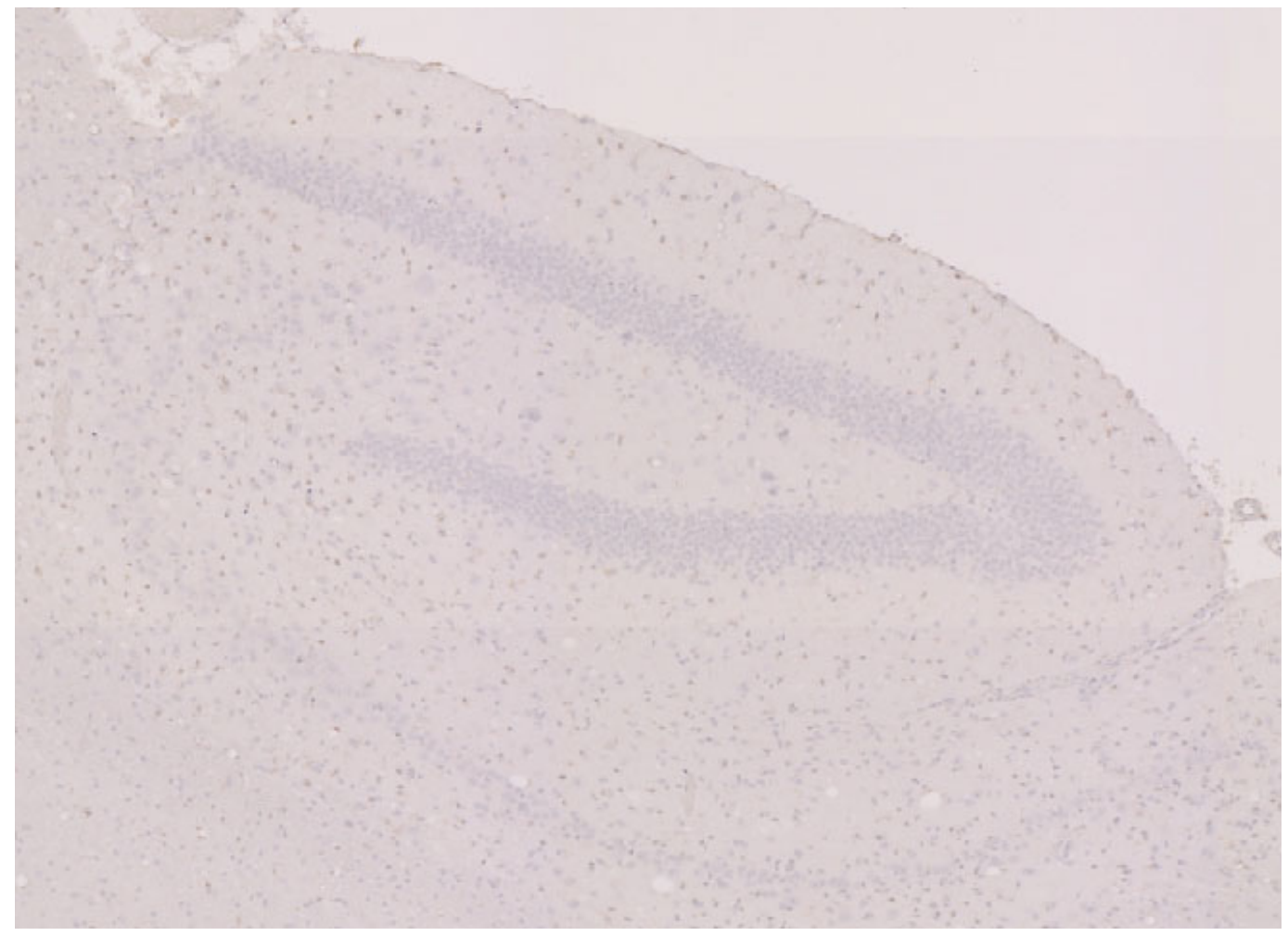

C

inoculum	preparation	capsid	payload	DPI
healthy brain extract	AAVX	9P31	EGFP	196

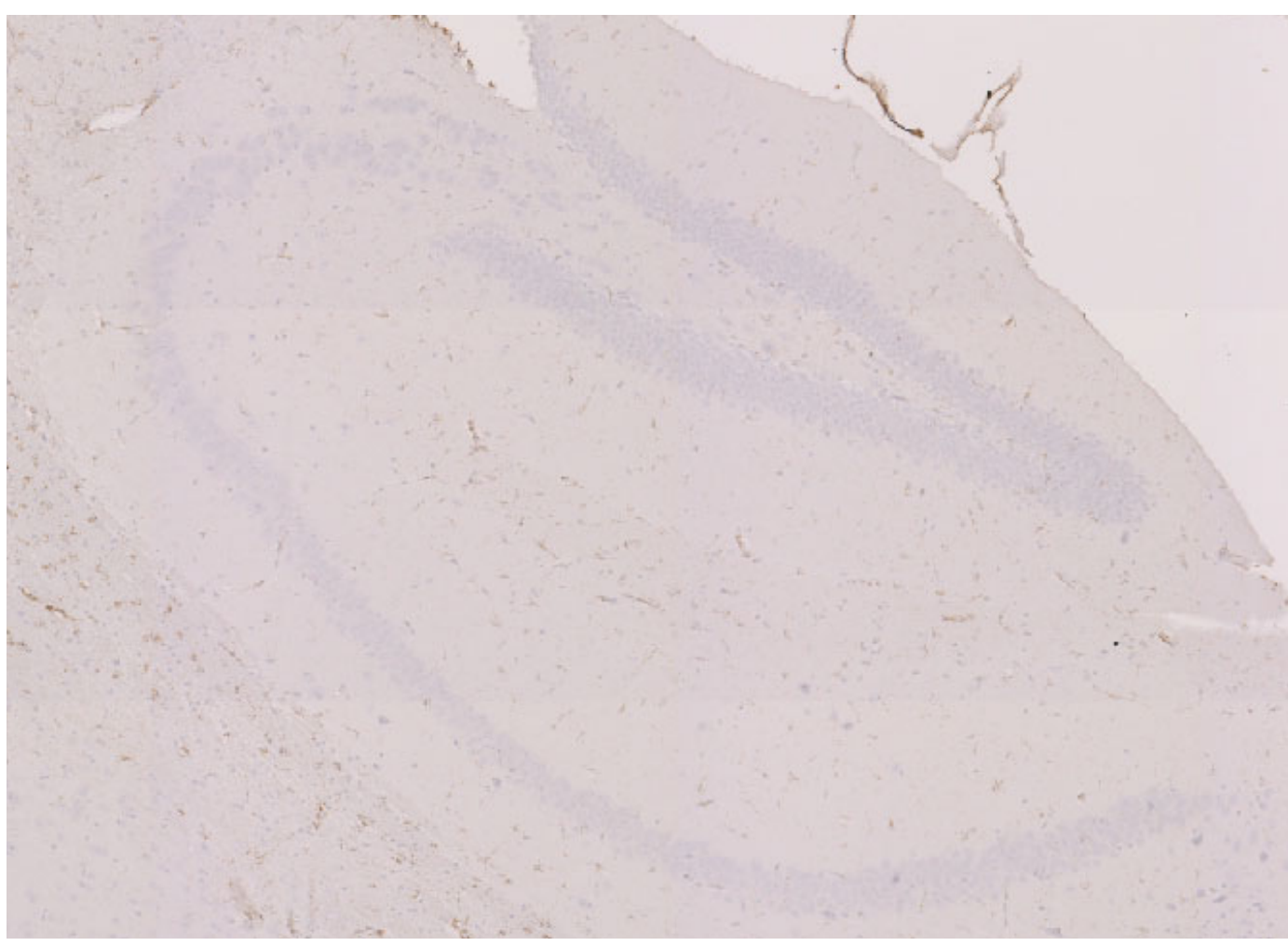

D

inoculum	preparation	capsid	payload	DPI
healthy brain extract	AAVX	9P31	EGFP	196

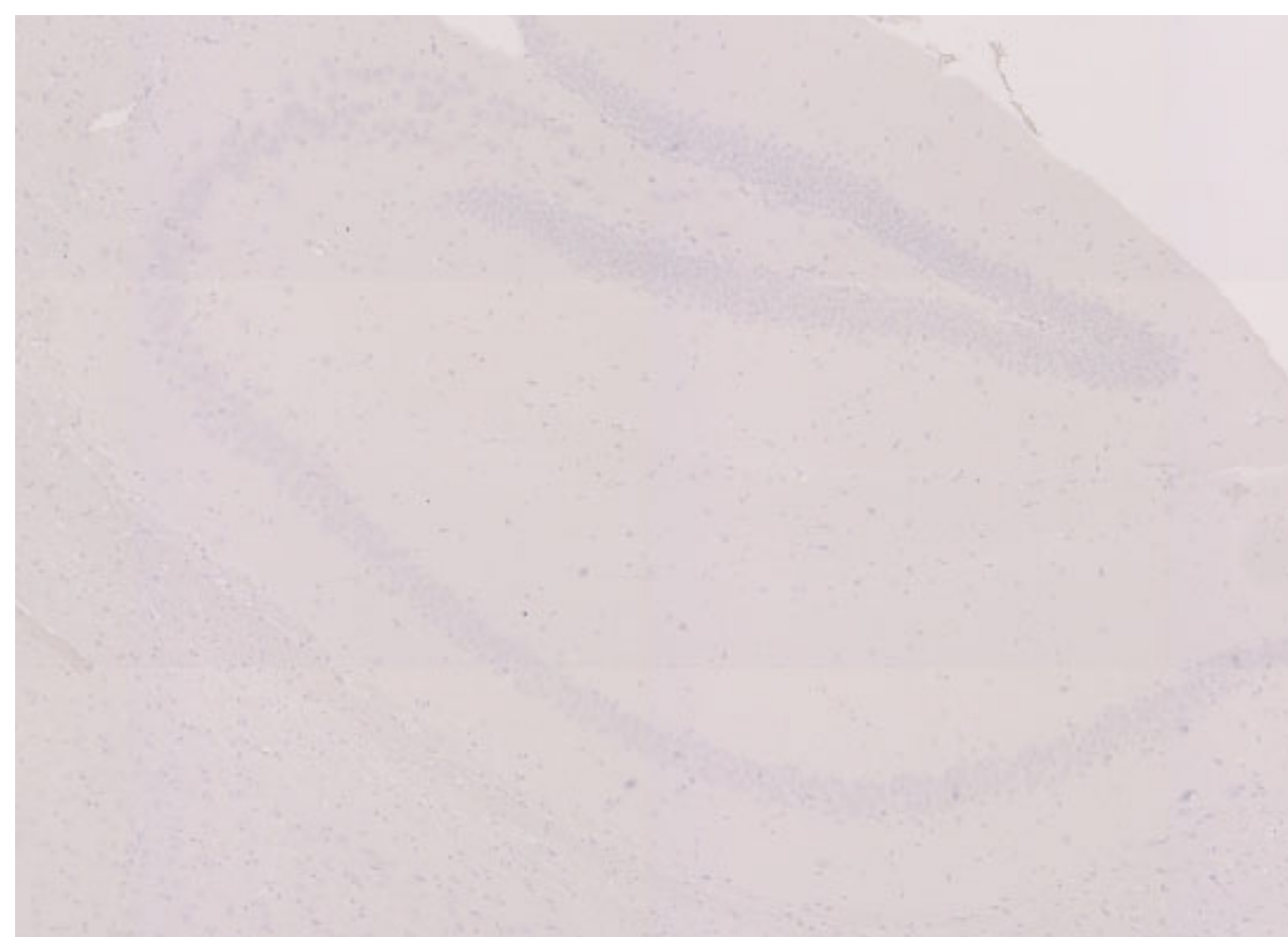

E

inoculum	preparation	capsid	payload	DPI
RML	AAVX	9P31	Bv*Prnp*^{V127}ΔGPI	196

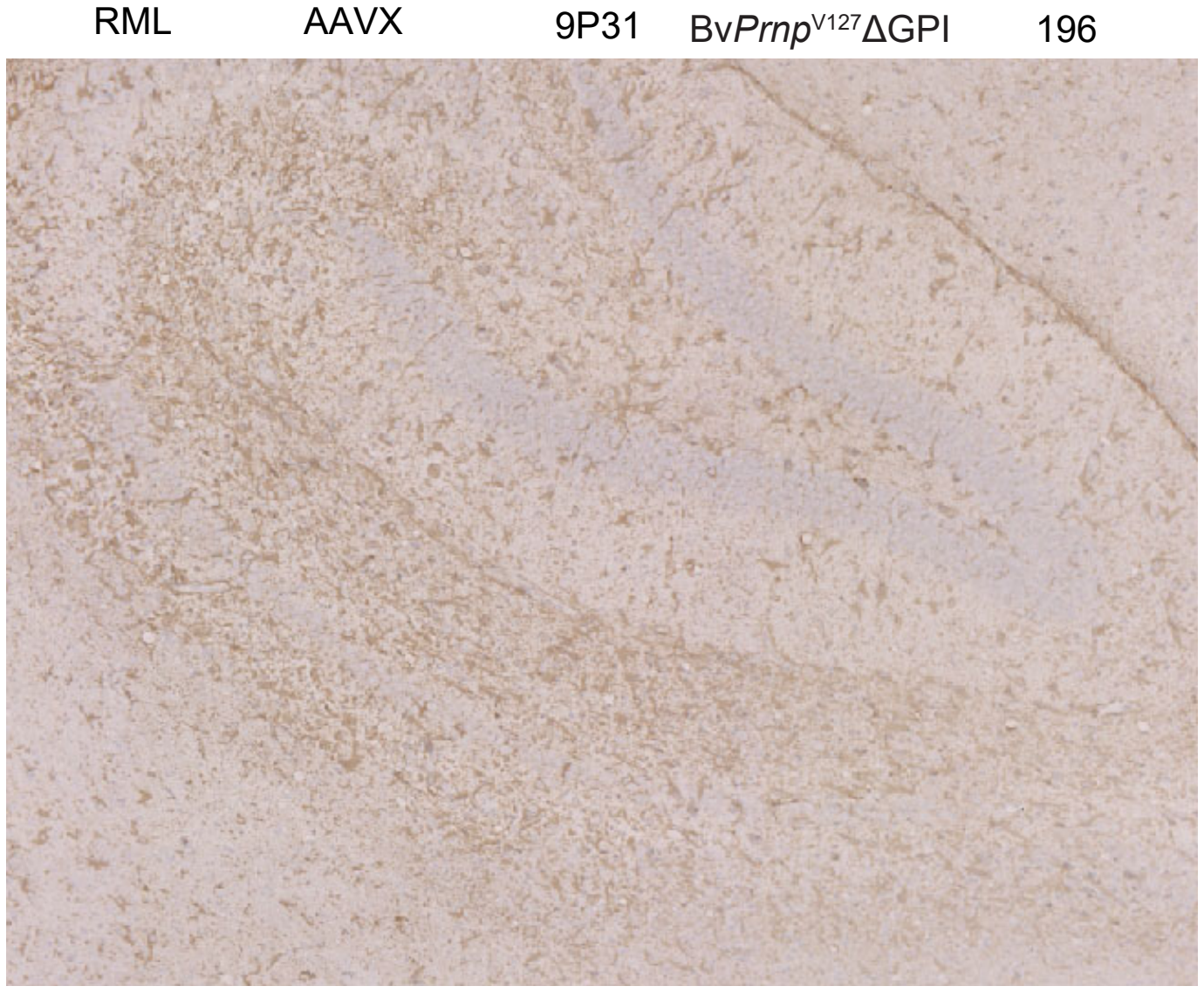

F

inoculum	preparation	capsid	payload	DPI
RML	AAVX	9P31	Bv*Prnp*^{V127}ΔGPI	196

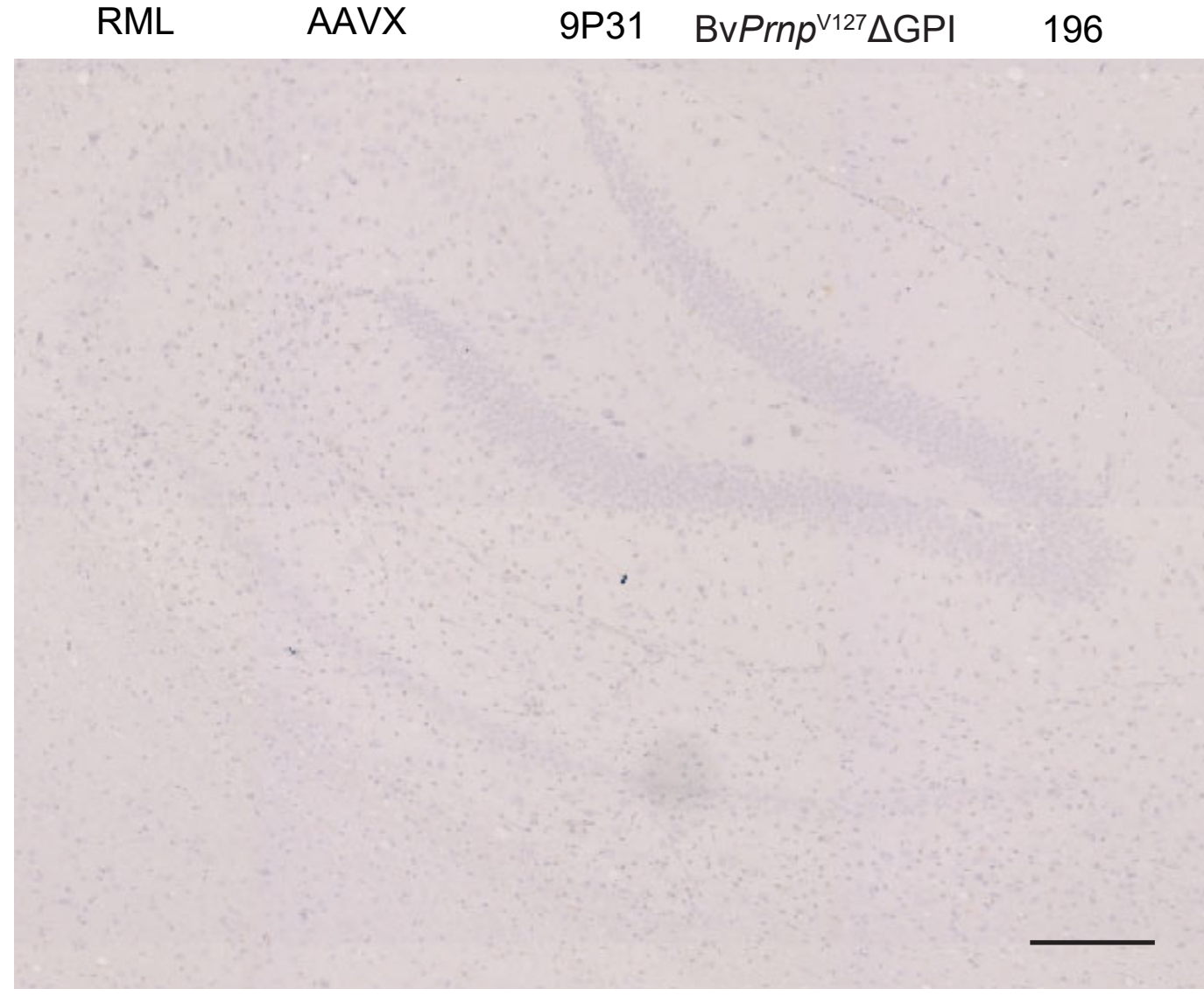

Supplement: Supplementary file 2 [file ppat.1014124.s002.pdf]
