# Supplementary material for: A self-complementary recombinant adeno-associated virus vector coding for an anchorless prion protein carrying the G127V mutation extends survival in a rodent prion disease model
Source: PLoS Pathog. 2026 Aug 4;22(8):e1014124. doi: 10.1371/journal.ppat.1014124 (PMC13460743; doi:10.1371/journal.ppat.1014124)

S1 Raw

Figure 1

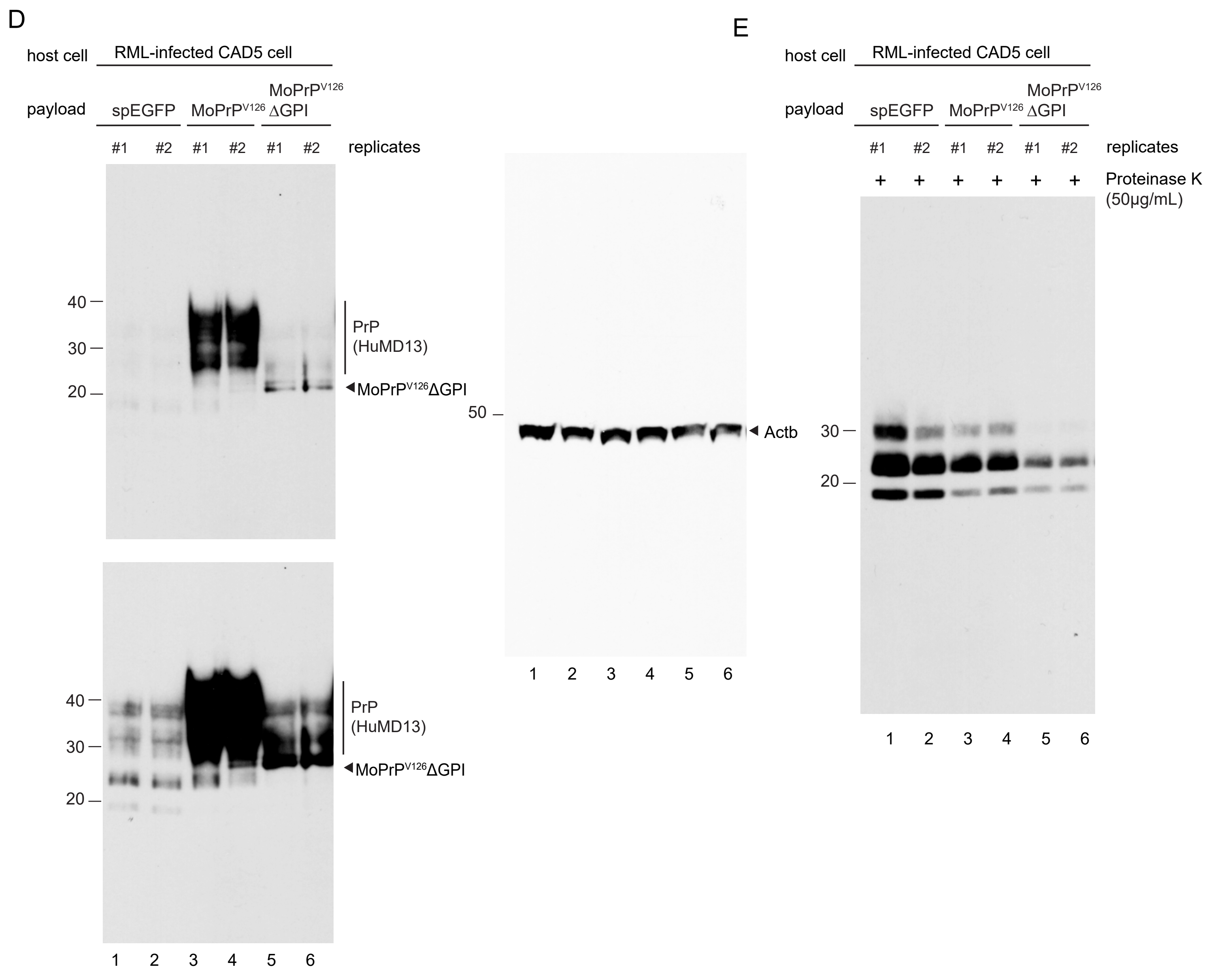

Figure 3

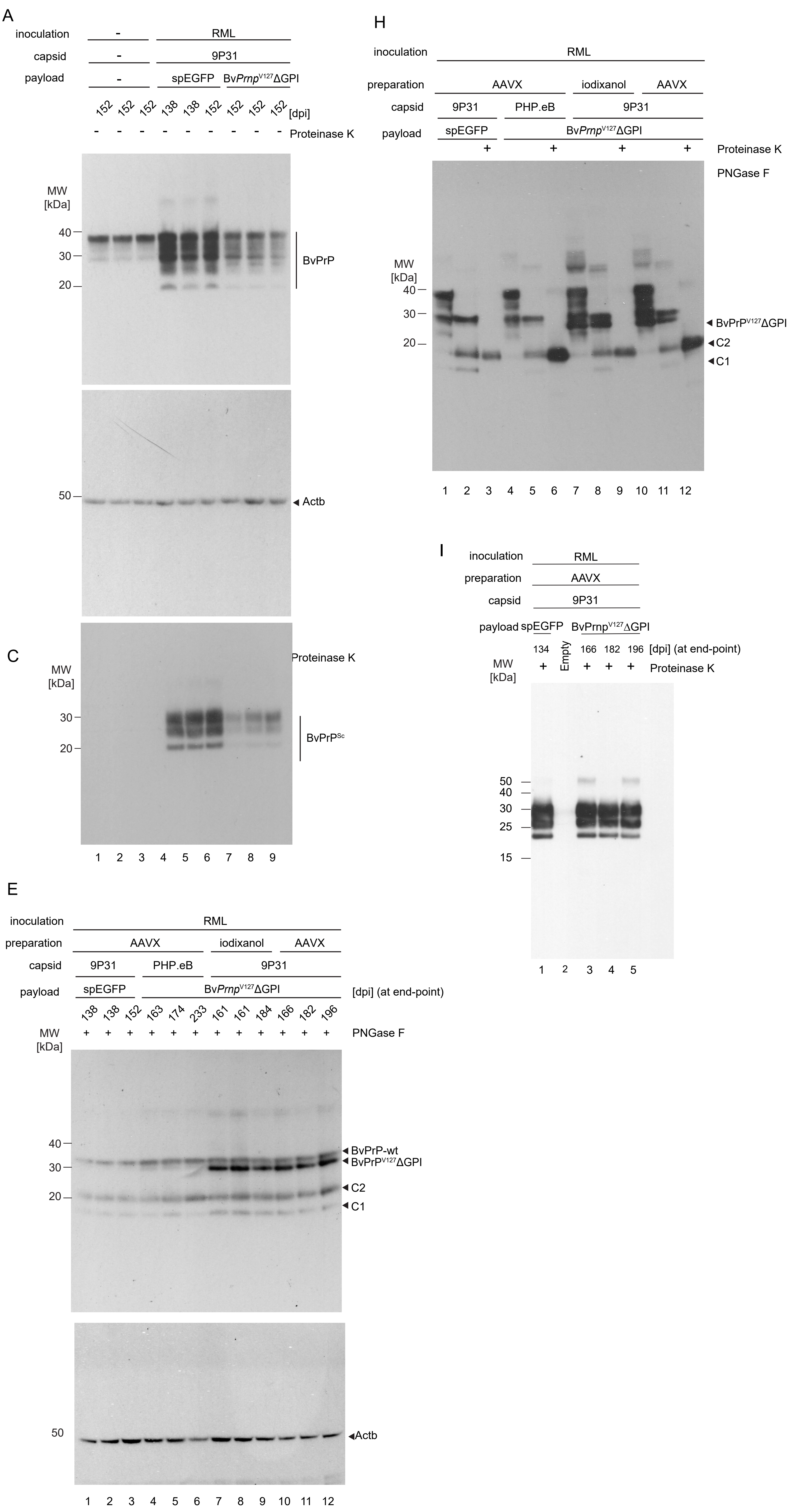

Figure 4

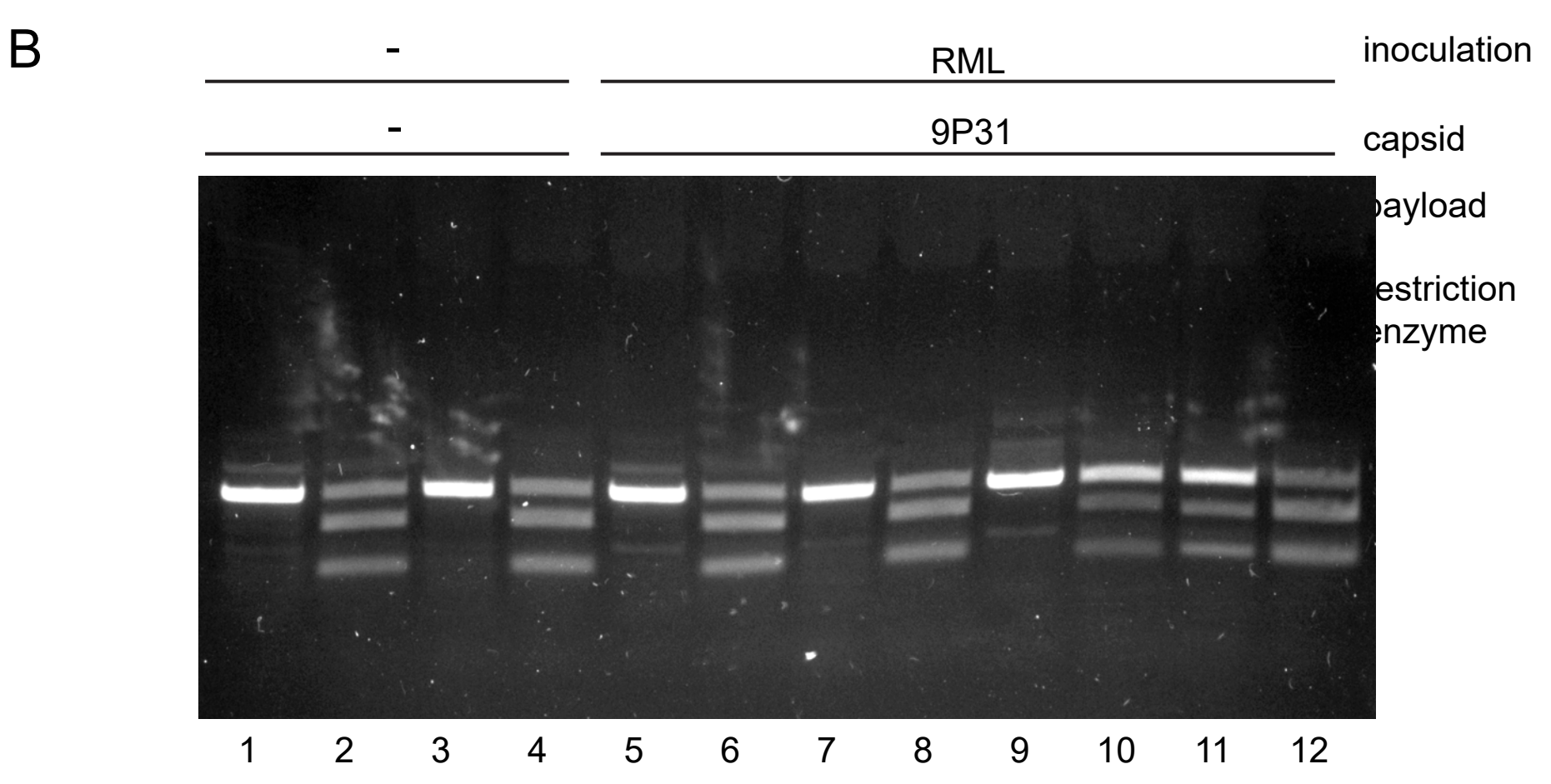

Figure 5

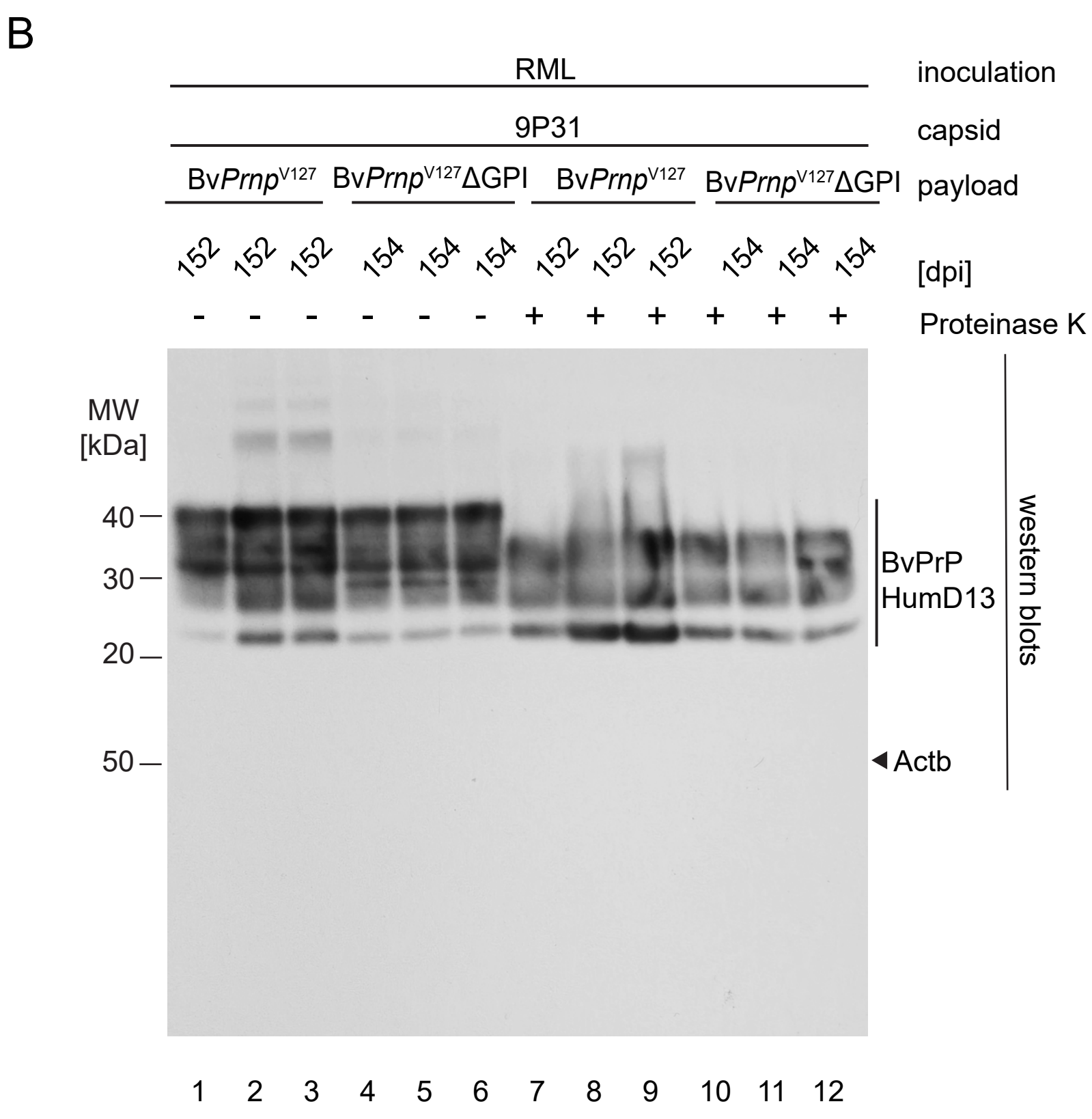

S1 Figure

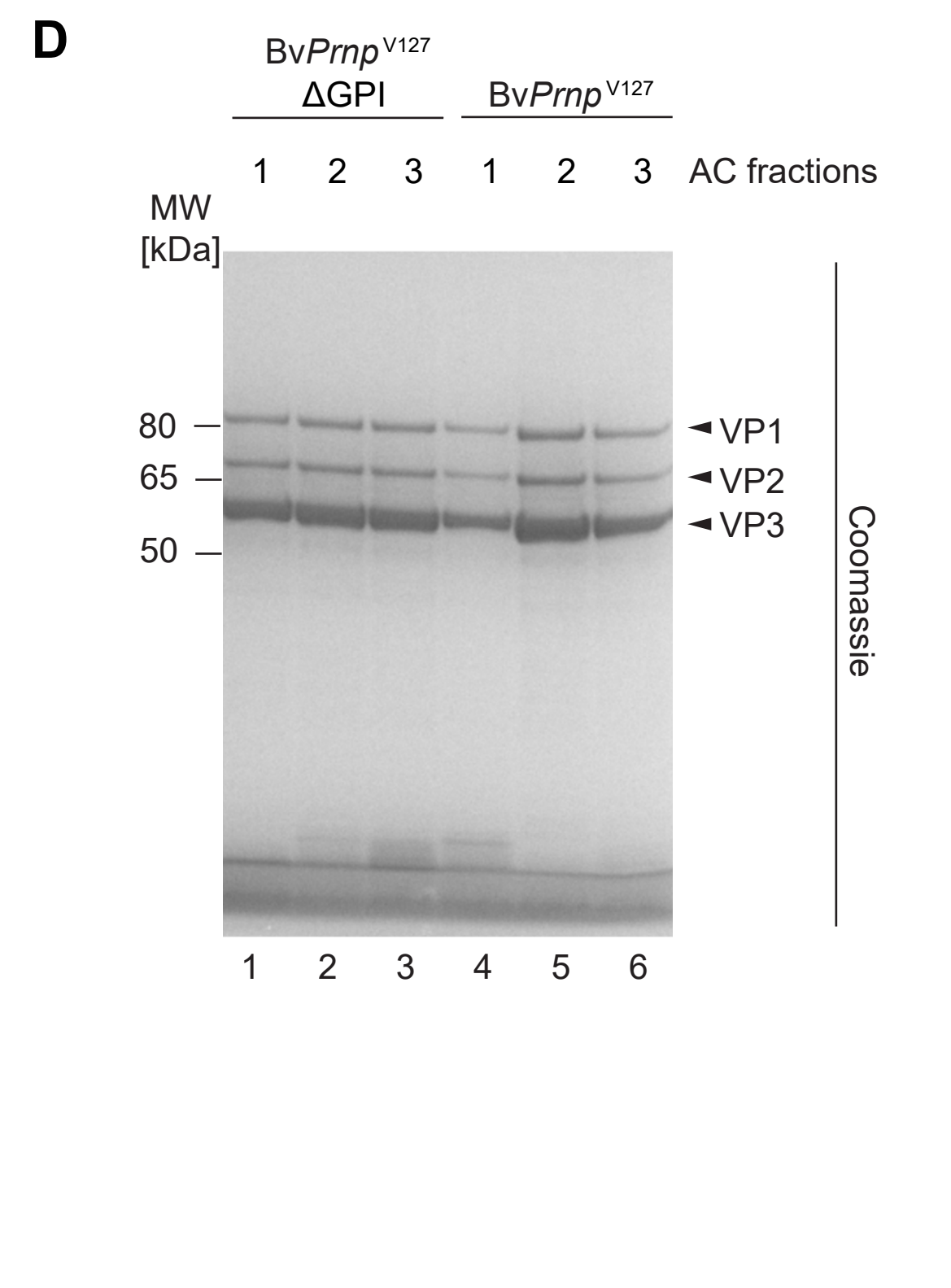

Supplement: Supplementary file 3 [file ppat.1014124.s003.pdf]
